# Nighttime human-supervised drone-delivered automated external defibrillators: a quasi-experimental mixed-methods feasibility study

**DOI:** 10.1016/j.resplu.2026.101457

**Published:** 2026-09-04

**Authors:** C.K.J. Chan, S.Y.J. Ho, A. Yip, F.L.N. Tung

**Affiliations:** a School of Nursing and Health Sciences, Hong Kong Metropolitan University, Hong Kong; b S.K. Yee School of Health Sciences, Saint Francis University, Hong Kong

**Keywords:** Out-of-hospital cardiac arrest, Automated external defibrillator, Unmanned aerial vehicle, Drone, Nighttime operations, Human factors, Mixed methods

## Abstract

**Aim:**

To evaluate the feasibility, safety, and operational challenges of human-supervised flight drone-delivered automated external defibrillator (AED) operations during nighttime for out-of-hospital cardiac arrest (OHCA), using a convergent mixed-methods design integrating quantitative performance metrics with qualitative human factors analysis.

**Methods:**

A quasi-experimental study with 24 controlled flights (12 daytime, 12 nighttime) was conducted in a semi-urban area using a DJI M30T drone carrying a lightweight CellAED (480 g) over 877 m. Advanced-rated paramedic drone pilots executed human-supervised flight flights under extended visual line of sight (EVLOS), using automated waypoint navigation and manual final approach and landing. protocols. Quantitative data included flight times and safety incidents, analyzed using paired *t*-tests. Qualitative data from semi-structured pilot interviews were analyzed thematically using the SHELL (Software-Hardware-Environment-Liveware-Liveware) human factors framework. Integration occurred through convergent triangulation comparing quantitative duration metrics with pilot-reported operational themes.

**Results:**

All 24 flights completed successfully without safety incidents or obstacle-avoidance triggers. Mean flight times were comparable (day: 136.00 ± 8.71 s vs. night: 139.92 ± 14.16 s; paired mean difference 3.92 s, S.D. 16.49, *p* = 0.43, Cohen’s *d* = 0.24). Qualitative analysis identified eight major themes: night-mode interface challenges, GPS routing reliability, infrared sensor performance, pilot fatigue and stress, communication coordination, cognitive workload, landing zone conditions, and regulatory constraints. Triangulation of quantitative and qualitative findings showed that despite comparable flight performance, pilots reported significantly increased cognitive demands and environmental complexity during nighttime operations.

**Conclusion:**

Under controlled, early-night, open-area conditions, all human-supervised flight drone-delivered AED operations missions were completed without observed safety incidents. However, successful nighttime implementation requires addressing pilot workload, interface design, and operational protocols. These findings support development of 24/7 drone AED services while highlighting critical human factors considerations for sustainable operations.

## Introduction

Out-of-hospital cardiac arrest (OHCA) remains a leading cause of mortality worldwide, with survival rates typically ranging from 8% to 10% despite advances in emergency medical systems.^1^ For patients with a shockable rhythm, early defibrillation is a critical determinant of survival and operates within a broader chain of survival that includes prompt recognition, activation of EMS, and high-quality CPR. —Each minute of delay reduces survival probability by approximately 6–7%.^2^, ^3^ This temporal urgency is particularly acute during nighttime hours in rural residential areas when traditional public-access AED infrastructure is often not available and emergency medical services (EMS) response times may be prolonged.^4^, ^5^

Unmanned aerial systems (UAS) for AED delivery have emerged as a transformative innovation.^6^ Recent real-world studies have demonstrated technical feasibility and safety of drone-delivered AEDs in urban settings in day time.^7^, ^8^, ^9^, ^10^, ^11^ A prospective Danish study of 76 suspected OHCAs achieved successful AED delivery in all 16 eligible cases with median response time of 4:47 min.^7^ These studies highlighting the importance of strategic deployment. Simulation studies have confirmed bystander acceptability and usability.^8^, ^12^ Schierbeck et al. (2023) show AED delivery by drone faster than ambulance arrive in 67% cases.^9^

However, most reported drone AED services and feasibility studies operate have been limited to daylight hours operation. Consequently, the 35.5–53.4% of OHCAs occurring during overnight periods may not benefit from drone-delivered AED coverage.^7^, ^13^ It representing an important gap in current evidence and operational capability. This limitation stems from airspace regulatory restrictions, technical constraints, and unvalidated assumptions about nighttime flight safety. Scholz et al. (2023) demonstrated that automated beyond visual line of sight (BVLOS) flights for nighttime AED delivery are technically viable using infrared camera, with comparable mission times between day and night operations.^14^ Despite this contribution, significant knowledge gaps persist regarding human-supervised flight. flight operations and human factors influencing pilot performance during nighttime missions.

Understanding these human factors in nighttime drone AED service is essential because real-world drone AED systems require human oversight for safety–critical decisions, especially landing zone selection or confirmation. A previous study of a fully automated drone AED delivery reported defined as the horizontal displacement from the prespecified landing target of 122 cm.^15^ Dispatcher stress and cognitive workload have been identified as operational concerns in daytime systems.^7^ Dispatcher uncertainty about when to instruct bystanders to retrieve drone-delivered AEDs has been documented. Additionally, bystander reluctance to leave patients unattended poses Behavioral challenges to AED retrieval.^8^, ^12^, ^16^, ^17^, ^18^, ^19^ These human factors must be systematically evaluated,^20^ especially under nighttime conditions where visual cues are diminished and cognitive demands for dispatcher may be amplified.

## Methods

### Study design

This study employed a convergent mixed-methods design^21^, ^22^ and reported according to the Good Reporting of A Mixed Methods Study (GRAMMS) framework.^23^ We evaluated the drone-AED delivery operations in a semi-urban setting by combining: (1) a quasi-experimental quantitative design comparing daytime versus nighttime flight performance, and (2) a qualitative approach exploring pilots’ experiences and operational challenges. Qualitative data were analysed with the SHELL (Software-Hardware-Environment-Liveware-Liveware) human factor engineering framework.^24^ Integration occurred through triangulation, comparing quantitative duration data with pilot-reported themes to identify convergence (agree), divergence (disagree), and expansion as complementarity insights across quantitative and qualitative strands. This design was selected to capture both objective performance metrics and subjective operational realities. To assessed the feasibility of nighttime operations while identifying operational complexities and human factors relevant to the development of sustainable 24-hour drone-AED services.

### Setting, participants and ethical considerations

The study was conducted at High Island Reservoir, Hong Kong (semi-urban area), chosen for its controlled environment devoid of light pollution, enabling assessment of nighttime operations without urban visual interference. The flight route included trees and other vegetation, utility poles and overhead cables, and uneven terrain; however, no buildings were present along the reservoir route. Twelve healthcare professionals (doctors, nurses, paramedics) holding advanced-rated Hong Kong Civil Aviation Department drone qualifications were recruited using purposive sampling. They were selected to model a potentially cost-conscious drone-AED service in which medical dispatchers or other healthcare personnel with appropriate drone qualifications may undertake drone operation as a secondary role rather than employ full-time specialist pilots. This approach enabled examination of operational experiences from a healthcare perspective, while recognising that the priorities, situational awareness, and decision-making of healthcare professionals who fly drones part-time may differ from those of dedicated professional drone pilots. All 12 participants provided written informed consent before participation, including consent for audio recording of the focus group. Ethical approval was obtained from Hong Kong Metropolitan University Human Research Ethics Committee (Ref: HE‑RGC2025/NHS02) and operational clearance from Hong Kong Civil Aviation Department (Permit: AOP/2412‑02). Data were anonymized and stored on encrypted USB drive. No patient data or real emergency scenarios were involved.

### Quantitative strand: flight performance evaluation

#### Equipment and procedures

We utilized a DJI M30T drone (3.9 kg) equipped with infrared camera, night vision capabilities, and high-intensity external strobe lights carrying a lightweight CellAED (480 g). All flights followed a standardized 877‑m route as Fig. 1, under extended visual line of sight (EVLOS) protocols, using automated waypoint navigation and manual final approach and landing. Safety visual observer positioned at landing observation points. In standard RGB camera mode, the image was too dark for reliable identification until infrared mode was activated, while the drone spotlight illuminated the ground during landing to confirm clearance.

**Fig. 1:**
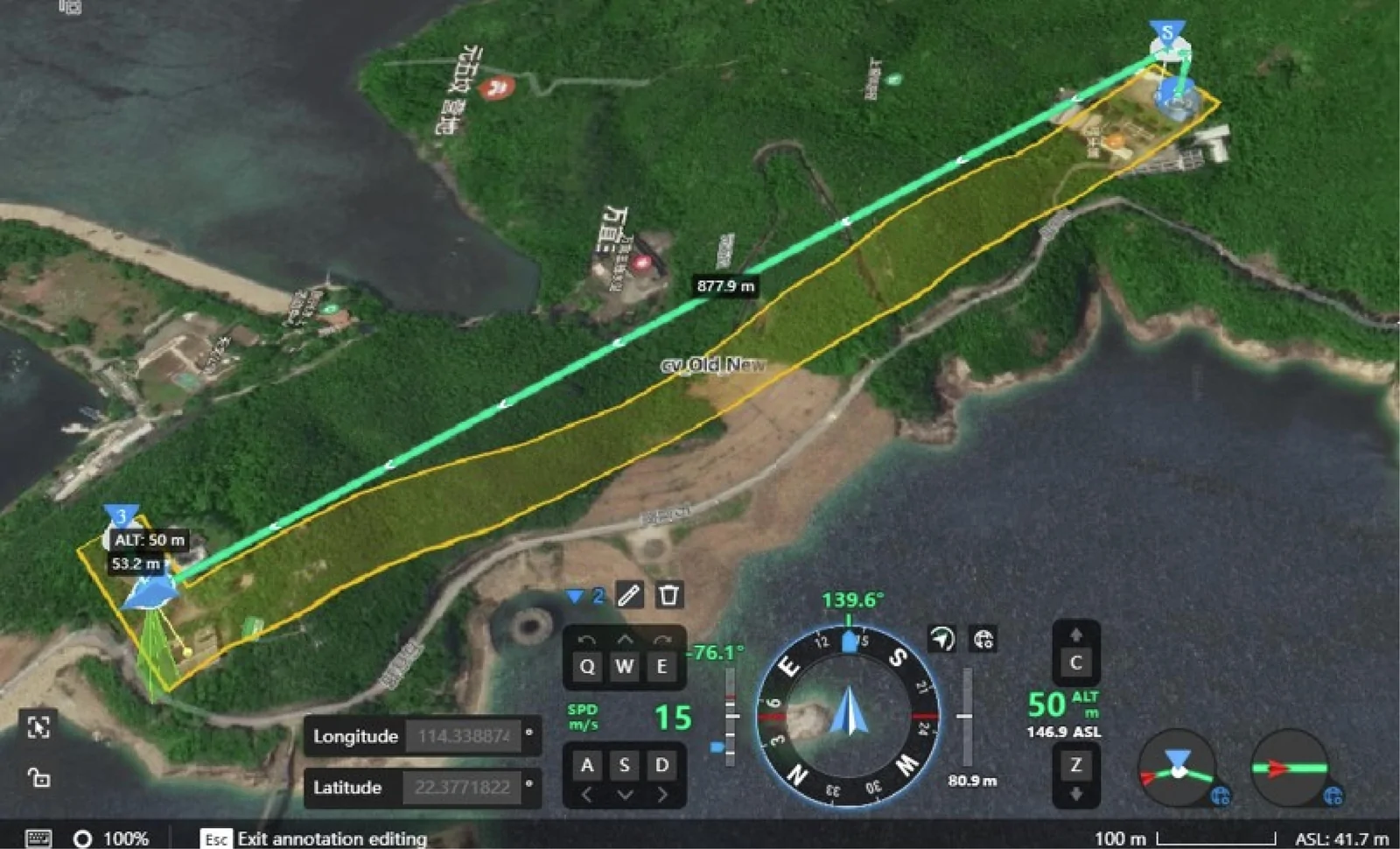
**Drone flight path, airspace risk, ground risk assessment for simulated scenario by DJI Flight hub 2 automated flight**.

Twenty-four controlled flights were conducted: 12 daytime (1400–1600 h) and 12 nighttime (1930–2100 h) within a single day, one each by each pilot within a single day at April 2025. The order of conditions was fixed and non-randomized. Each participant completed the daytime condition before the nighttime condition in the same participant order. Flight parameters included: altitude 30–50 m AGL (Above-Ground-Level), speed 6 m/s predetermined landing zone coordinates within a predefined 1 m × 1 m landing pad. No winch was used and no simulated recipient retrieved the AED.

Environmental conditions were monitored as wind <12 m/s and no precipitation. The illuminance level was 0.11–0.15 lx when the light source was switched off, as measured using a photometer at 3 cross session 19:30, 20:15 and 21:00.

#### Data collection and measures

Primary outcome was total flight time (seconds) from takeoff to landing and AED delivery, measured using drone telemetry logs synchronized with ground timing systems (±0.5 s precision). Secondary outcomes included obstacle avoidance system activations, GPS signal loss events, return‑to‑home triggers, and critical safety incidents.

#### Statistical analysis

Total 24 flights (12 daytime, 12 nighttime) was pragmatically determined based operational feasibility, the availability of qualified participants, and simple size used in related prior drone flight studies 16–20 flights^7^, ^14^ and Julious’ recommendations for pilot feasibility studies.^25^ Normality was assessed using Shapiro–Wilk tests. Flight time differences were analyzed using paired t‑tests. Statistical significance was set at *α* = 0.05. Effect sizes were calculated using Cohen’s *d*. Analysis was conducted using SPSS version 30.

### Qualitative strand: pilot experience evaluation

Data Collection: One 60 min semi-structured focus group interviews were conducted on the same day with all twelve pilots immediately after flight completion. The focus group was planned, audio-recorded with consent. The study-specific interview guide was developed and reviewed by the research team using the established SHELL aviation human factors framework^24^: Software (interface/systems), Hardware (equipment), Environment (external conditions), and Liveware–Liveware (human–human interactions). The protocol allowed clarification or follow-up contact if important issues remained unresolved. No additional interviews were undertaken because the group generated sufficiently detailed data relevant to the prespecified research question.

#### Research team and reflexivity

Interviews were conducted by AY (female, nurse academic with qualitative research training) and CKJ (male, nurse academic with drone and human factors research experience). Both interviewers documented their assumptions (for example, that night‑time operations would be more cognitively demanding) and reflected on how these might shape interactions and data interpretation. AY facilitated focus groups while CKJ observed and took field notes.

Open-ended questions explored experiences flying at night compared to daytime, operational challenges encountered, effects of nighttime conditions on decision-making, and support or modifications needed for nighttime operations. By the end of the focus group, the facilitators observed substantial repetition of responses and no new issues arose within the prespecified SHELL domains. Given that only one focus group was conducted, formal saturation across multiple data-collection sessions was not assessed. Reflexive notes were maintained throughout, and preliminary themes were presented to the research team (FTLN and JHSY) for critical feedback to minimize interpretive bias and enhance credibility.

#### Data analysis

Audio recordings were transcribed verbatim. Two researchers (CKJ and AY) independently coded all transcripts line‑by‑line in NVivo 15 using Braun and Clarke’s six‑phase framework^26^: familiarization, initial coding, theme development, theme review, definition, and reporting. Researchers met to compare coding, resolve discrepancies through discussion, and agree on a shared coding tree. Themes were organized within the SHELL framework domains. Credibility was supported through triangulation with telemetry data, field notes, and peer debriefing within the research team.

### Mixed methods integration

Integration occurred through convergent triangulation.^27^ Quantitative flight time data were compared with qualitative themes related to operational complexity, cognitive workload, and environmental challenges. Joint display and integrative interpretations were developed collaboratively by CKJ, AY, FTLN, and JHSY during three iterative team meetings, ensuring both quantitative and qualitative perspectives were represented.^28^ Convergence (agreement), divergence (disagreement), and expansion (complementary insights) were identified and interpreted. This integration addresses the research question: “How do objective flight performance metrics align with subjective pilot experiences of operational complexity during nighttime medical emergency operations?”

## Results

Objective flight outcomes showed no statistically significant differences between daytime and nighttime operations. However, qualitative findings indicated that nighttime flights involved greater perceived workload, reliance on onboard sensors, and operational complexity.

### Quantitative findings: flight performance

All 24 flights (12 daytime, 12 nighttime) were completed successfully without safety incidents, obstacle avoidance activations, GPS signal loss, or emergency procedures. Mean flight times were 136.00 ± 8.71 s (day) vs. 139.92 ± 14.16 s (night). The mean flight time difference of 3.92 s (2.9% increase) was not statistically significant (*t*(11) = 0.81, *p* = 0.43) with small effect size (Cohen’s *d* = 0.24). All flights adhered to predetermined flight paths with no deviations. Wind conditions were similar and negligible for drone (designed for 12 m/s): mean wind speed 4.2 m/s (daytime) vs. 3.8 m/s (nighttime). No precipitation occurred on the research date.

### Qualitative findings: pilot experiences

Thematic analysis identified eight major themes organized within the SHELL framework as below 4 themes, details in Table 1.

**Table 1:** SHELL framework themes from pilot interviews with representative quotations.

**SHELL domain**	**Theme and representative quotation**
Software	**Night-mode interface challenges:** “The screen brightness automatically adjusts but it's still hard to see details at night. I found myself squinting more and decrease brightness manually.” (P03)
	**GPS routing reliability:** “GPS works fine but at night I double-check waypoints more, because everywhere is dark from camera (without Infrared) you can't visually confirm landmarks as easily.” (P08)

Hardware	**Infrared sensor performance:** “The thermal camera is amazing for seeing obstacles but the image outline is different from daylight—takes mental adjustment to interpret what you're seeing.” (P02)
	“At night it feels almost impossible to fly safely without an infrared camera, because you lose most visual cues and have to rely on thermal images to detect obstacles and people.” (P08)
	**Strobe light and visibility:** “The strobe light is mainly useful during landing and for rescuers to locate the drone more easily; during normal flight it doesn’t help much with navigation, but it helps others see where the drone is.” (P08)

Environment	**Environmental complexity:** “Night operations are quieter but also more isolating. Depth perception changes without natural light, especially during landing.” (P06)
	“Flying in the daytime to familiarise myself with the environment is very helpful for night flying, because I already have a mental picture of key landmarks and potential hazards.” (P01)
	“The 3D model in the software platform increases my confidence in obstacle avoidance and maintaining the required altitude, because I can visualise the terrain and structures more clearly than with a flat map.” (P02)
	**Landing zone assessment:** “Judging surface conditions at night relies heavily on thermal imaging and strobe lights. I feel less confident about unexpected obstacles.” (P04)
	“The system can’t be fully automated, because the landing site still needs human confirmation to judge safety—sensors and maps can’t always detect small obstacles or sudden changes on the ground.” (P10)

Liveware	**Pilot fatigue and stress:** “Night flights require more concentration. After three consecutive night missions I felt mentally drained in a way I don't during daytime.” (P10)
	**Cognitive workload:** “There's more to process at night—checking instruments more frequently, relying on sensors versus direct visual confirmation, heightened alertness for anything unusual.” (P11)

#### Software themes

Pilots reported automatic brightness dimming reduced screen visibility, requiring manual overrides that increased cognitive load. GPS routing was perceived as reliable but prompted increased verification behaviours due to reduced visual landmark confirmation.

#### Hardware themes

Infrared sensor performance was valued for obstacle detection but required cognitive adaptation. Pilots described a “learning curve” in interpreting thermal imagery compared to natural vision, particularly for distinguishing surface textures and assessing landing zone suitability.

#### Environment themes

Environmental complexity increased at night due to reduced depth perception and ambient lighting variability. Pilots noted greater reliance on artificial lighting (strobe lights, drone LEDs) and instrument readings. Landing zone assessment was more challenging, requiring longer hover times for thermal scanning and surface evaluation.

#### Liveware themes

Most pilots (*n* = 8) reported heightened addition mental effort during nighttime flights, describing increased monitoring frequency, slower decision-making, and greater post‑flight fatigue.

### Mixed methods integration: joint display

The critical integration insight is divergence between objective performance and subjective experience: while flight times and safety outcomes were no difference was found, pilots consistently reported significantly increased cognitive demands, stress, and workload during nighttime operations. This divergence suggests that no statistically significant difference in flight time performance was achieved through compensatory pilot effort—increased monitoring, verification behaviors, and cognitive resources—rather than true operational equivalence. Details in Table 3.

**Table 2:** Implication for 24/7 Drone AED services.

Interface optimization	Night‑mode displays should be designed specifically for nighttime use rather than simply dimming daytime interfaces. Enhanced thermal image processing, pre‑configured night profiles, and adaptive display systems are needed.
Training and competency	Nighttime operations require specialized training beyond daytime proficiency. Training programs should include nighttime‑specific scenarios, thermal imaging interpretation modules, and assessment of night competency separately from daytime skills.
Workload management	The finding that equivalent performance required increased compensatory effort suggests current systems place unsustainable demands on pilots during nighttime. Automation of routine monitoring tasks, decision support systems for landing zone assessment, and enhanced sensor integration could reduce manual workload. Operational protocols should limit consecutive nighttime missions and mandate rest periods, recognizing fatigue risk.
Regulatory framework	Current regulations in many jurisdictions restrict nighttime or BVLOS operations. Our safety data (zero incidents across 12 nighttime flights) supports regulatory reconsideration of blanket nighttime restrictions. However, regulations should mandate night‑specific training, equipment standards (infrared capability, lighting), and operational protocols rather than simply extending daytime authorizations.

**Table 3:** Joint display of quantitative flight outcomes and qualitative findings for nighttime drone-delivered AED operations.

**Quantitative finding**	**Related qualitative theme**	**Integration type**	**Integrated interpretation**	**Operational implication**
Daytime 136.00 ± 8.72 s; nighttime 139.92 ± 14.16 s; paired difference 3.92 ± 16.49 s; *p* = 0.43	Greater monitoring, verification and cognitive workload at night	Divergence	Mission duration did not differ significantly, but pilots described greater compensatory effort during nighttime flights.	Evaluate workload-reducing interfaces and operational protocols before repeated nighttime deployment.
No observed safety incidents, GPS loss, return-to-home activation or obstacle-avoidance activation	Reduced visual cues and greater reliance on infrared imaging and the landing spotlight	Expansion	Event-free missions occurred under favorable, open, early-night conditions and do not establish general operational safety.	Test adverse weather, later-night periods, repeated missions and more complex environments.
Ambient illuminance was 0.11–0.15 lx when other light sources were off	Standard camera imagery was not interpretable until infrared mode was activated	Convergence	Objective low-illumination conditions corresponded with reported dependence on sensor-based information.	Include infrared capability, appropriate lighting and night-specific competency requirements in future protocols.

## Discussion

This mixed-methods study observed that under controlled, early-night, open-area conditions, all missions were completed without observed safety incidents.

The finding has important implications for sustainable 24/7 operations, as relying on sustained compensatory effort may lead to long‑term fatigue and performance degradation. However, qualitative analysis reveals that this equivalence masks significantly increased pilot cognitive workload, reliance on technological aids, and compensatory behaviors. The integration of quantitative and qualitative strands provides a more complete understanding than either method alone: nighttime operations are technically achievable but operationally more demanding, requiring system-level support to ensure sustainability. Our quantitative findings align with Scholz et al. (2023), who reported similar total mission times between day and night in automated BVLOS flights (day: 7:59 ± 2:27 min vs. night: 7:26 ± 2:29 min).^14^

Our study extends this work in three critical ways. First, we demonstrate feasibility in human-supervised flight operations, addressing the gap regarding human‑piloted systems that many jurisdictions require for EVLOS operations, especially for landing zone confirmation. Second, our semi‑urban setting contrasts with Scholz’s^14^ rural environment, providing evidence for nighttime operations in more populated areas where drone AED services may have greatest impact. Third, our mixed-methods approach reveals human factors invisible in purely quantitative assessments.

Our findings suggest that extending to nighttime coverage is technically feasible and safe but will require addressing pilot-specific factors including night operation interface design optimization, fatigue management protocols, and enhanced training for night‑specific scenarios. The Danish real-world implementation study by Jakobsen et al. (2025) identified dispatcher concerns about bystander distress and system usability.^7^ The simulation study by Smith et al. (2024) emphasized the importance of dispatcher support and AED usability for lone bystanders^8^; similarly, our findings highlight that human factor engineering pilot support systems including enhanced visual displays and decision aids are essential for nighttime operations.

Human factors analysis using the SHELL framework^24^ revealed challenges spanning all domains. Software interface issues were manageable but added cognitive load. Hardware reliance on infrared sensors was effective but required adaptation. Environmental complexity necessitated compensatory strategies. Liveware factors (fatigue, workload) emerged as the most significant concern for sustainable operations. These findings align with broader aviation human factors literature demonstrating that night operations universally increase cognitive demands and error risk, requiring system‑level mitigations.

Medical qualifications are not a universal requirement for future drone pilots. However, clinically qualified operators may be beneficial when the pilot must also provide medical instructions or support emergency dispatch. Combining operational and clinical roles may be more cost-feasible than deploying separate pilots and healthcare professionals, while reducing communication and teamwork challenges.

### Implications for 24/7 drone AED services

Achieving sustainable 24/7 drone AED coverage requires moving beyond technical feasibility to operational sustainability. Our findings suggest critical system modifications as Table 2.

### Strengths and limitations

Strengths include the mixed-methods design providing both objective performance data and subjective operational insights, use of the validated SHELL framework for human factors analysis, controlled experimental conditions enabling causal inference about day/night differences, and participation of experienced healthcare‑pilots representing realistic operational contexts. The study suggests methodological rigor through multiple data sources and systematic integration procedures.

Limitations include one focus group and small sample size (*n* = 12 pilots, 1 flight per condition per pilot) limiting generalizability, although this sample size was considered adequate for feasibility assessment, it was not sufficient and aims to establish equivalence or detect a prespecified clinically meaningful difference. The use of the same participant sequence across both conditions is an internal-validity limitation, as it cannot fully eliminate period, fatigue, or learning effects. Testing between 19:30 and 21:00 represents early-night operations and may underestimate workload and fatigue during late-night or early-morning missions. The controlled environment lacks urban complexity (buildings, electromagnetic interference, light pollution), it may potentially underestimating real‑world nighttime challenges. Flights were conducted under favorable weather conditions; performance during adverse nighttime conditions remains unknown. The study assessed human-supervised flight EVLOS operations rather than full autonomous BVLOS flights, which may exhibit minor different human factors profiles. All pilots were healthcare professionals, not full‑time drone operators; experience level and professional background may influence nighttime performance. The quantitative performance result applies specifically to the DJI M30T–CellAED configuration (approximately 5 kg total weight) and should be interpreted primarily as workflow and human-factors testing. Although these findings may inform other configurations, heavier AEDs may require higher-payload aircraft, and payload capacity, endurance, mounting, and regulatory requirements require system-specific validation.

The study did not assess bystander interaction with nighttime‑delivered AEDs. No manikin or human recipient was placed beside the pad because minimum drone-person clearance requirements vary by jurisdiction and aircraft protective configuration. Therefore, this study does not propose any clearance distance. Controlled 877 m flights do not capture real‑world variability including unpredictable OHCA locations, bystander interactions, concurrent emergency service operations, and system integration challenges. Pilot interviews conducted by known research team members may have influenced responses toward positive reporting. Economic feasibility for scaled implementation was not assessed. Future research should simulate on conditions directly extending the nighttime question and failure: GPS loss, unexpected obstacles, adverse weather, extended late-night or early-morning operations, and repeated missions with cumulative fatigue.

## Conclusion

This mixed-methods study suggests that human-supervised flight drone-delivered AED operations during under controlled, nighttime, open-area condition are technically feasible and achieve no statistically significant flight performance to daytime operations. However, pilots reported greater cognitive workload and operational complexity at night. Technical capability alone is insufficient, sustainable 24/7 implementation will therefore require optimized pilot workload, system design, and operating procedures. Future research should evaluate these systems within real-world emergency-response workflows, failure and adverse weather.

## Declaration of AI use

An artificial intelligence assistant (ChatGPT version 5.5) was used to support grammar checking, wording refinement, transcript voice recording to text, and improvement of paragraph flow in this manuscript. The AI was not used for data analysis, or interpretation, and all scientific content, study design, and conclusions were conceived, verified, and approved by the authors.

## Data availability statement

Anonymized flight telemetry data and de‑identified interview transcripts are available from the corresponding author upon reasonable request and with appropriate ethical approval.

## CRediT authorship contribution statement

**C.K.J. Chan:** Writing – review & editing, Writing – original draft, Software, Resources, Project administration, Methodology, Investigation, Formal analysis, Data curation, Conceptualization. **S.Y.J. Ho:** . **A. Yip:** Writing – original draft, Methodology, Investigation, Formal analysis, Data curation, Conceptualization. **F.L.N. Tung:** Writing – review & editing, Methodology, Investigation, Formal analysis, Data curation.

## Funding

This study received no funding support. The authors would like to thank Hong Kong Metropolitan University (HKMU) for providing financial support for the Article Processing Charge (APC) associated with this publication.

## Declaration of competing interest

The authors affirm that there are no financial, personal, or professional conflicts of interest associated with this work. No financial support or benefits from commercial entities have been received that are directly or indirectly related to the research, analysis, or publication of this manuscript.
